# Water-Based Route for Dopamine and Reduced Graphene Oxide Aerogel Production

**DOI:** 10.1021/acsomega.3c05955

**Published:** 2023-11-29

**Authors:** Öznur Kavak, Barış Can, Erhan Bat

**Affiliations:** Department of Chemical Engineering, Middle East Technical University, Ankara 06800, Turkey

## Abstract

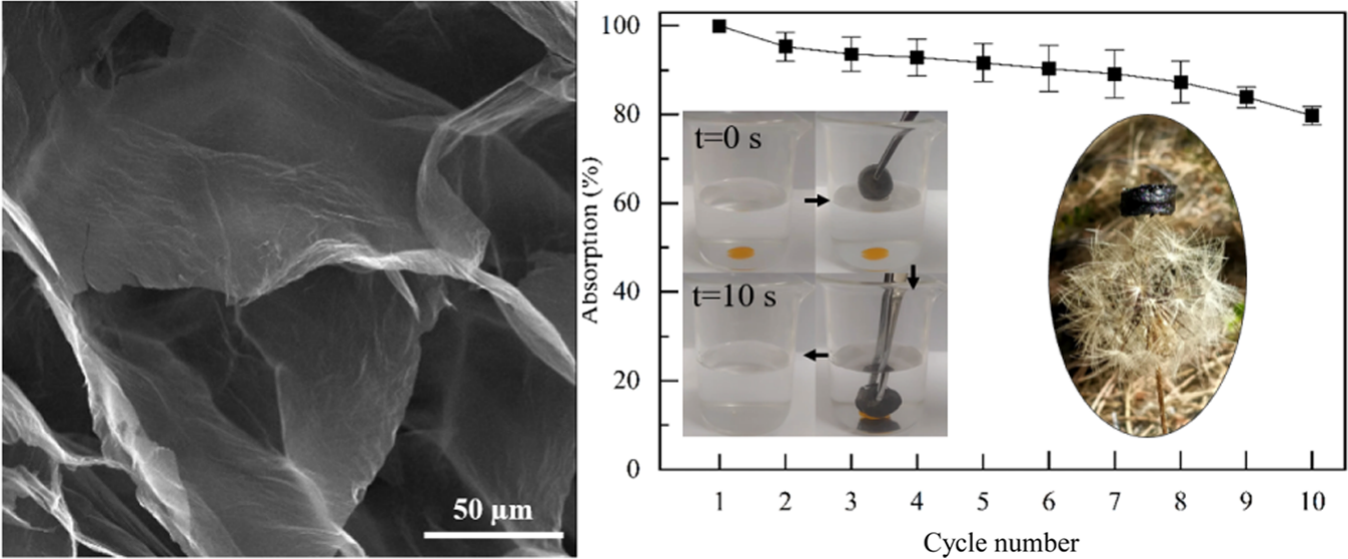

Water pollution caused by domestic waste oil and accidents with oil/organic spill needs immediate remediation, as such a pollution causes serious threats to health and the environment. Development of absorbent materials for the treatment of oil-polluted waters in a green and energy-efficient manner is highly desired. In this study, a green and simple strategy is proposed to prepare aerogels by hydrothermal reaction of graphene oxide (GO) dispersions using dopamine (DOPA) as the cross-linker. Concentrations of GO and DOPA were changed to determine their effects on absorption capacities. Aerogels produced had low densities ranging from 2.90 to 4.34 mg/cm^3^. Various organics, diesel oil, and sunflower oil were used to evaluate the absorption capacity of aerogels. It was observed that even with a mild thermal reduction at 150 °C, aerogels exhibited very high absorption capacities of up to 445 mg/mg. The produced aerogels showed high reusability (80%) and structural stability even after 10 absorption/desorption cycles. They possess great potential in oil/organic removal and water treatment based on their high absorption capacities and performances in separating organics/liquids from water.

## 1 Introduction

The increase in the frequency of oil spills during transportation, and/or drilling operations, and the discharge of chemicals into the sea attracted tremendous attention toward the resulting water pollution.^1−3^ According to a survey,^4^ between 2022 and 2023, approximately 213 million tons of vegetable oil (palm, sunflower, soybean, olive, coconut oil, etc.) were consumed in the world. Also, the amount of domestic waste oil, which joins to the wastewater eventually, has reached 100,000 to 700,000 tons/year in Europe.^5,6^ Most of the living creatures in coastal areas and undersea are affected negatively by the polluted water.^7^ Therefore, it is urgent to find effective solutions for the remediation of contaminated water. Many techniques have been used to address this issue, including centrifugation, filtration, in situ burning, etc. Among these techniques, the use of three-dimensional (3D) porous materials as sorbents comes to the forefront by providing effective removal of oil from the water, good recyclability, and oil recovery,^2,3^ hence eliminating the challenges such as low efficiencies, prolonged durations, or generation of secondary pollution.^8−11^

Aerogels, as absorbent materials, are very similar to hydrogels, and they can be obtained by removing water from the precursor hydrogel and replacing it with air using supercritical drying, freeze-drying, etc. Aerogels have constant mass and shape in their solid forms.^12,13^ They have open pore structure, low density, and high surface area, which also make them preferable in many applications as well as in oil–water separation. In order to increase the absorbance capacity as well as to increase the surface area of the aerogels, carbonaceous nanostructures such as carbon nanotubes and graphene derivatives can be integrated into the 3D structure of aerogels.^14−18^ These nanostructures also offer high mechanical strength, high aspect ratio, and high thermal and electrical conductivities in addition to their remarkable adsorption and separation performances.^2,19−22^

Graphene oxide (GO) has six carbon atoms present in the benzene ring that could provide an unpaired electron and aromatic sp^2^ domains that can allow GO to participate in a wide range of bonding interactions, enabling GO to have advantages in water purification and separation.^14,18,23−25^ As an oxygen-containing derivative of graphene, not only GO/rGO has many excellent properties (large mechanical strength, large specific surface area, high chemical stability etc.),^26^ but also it provides active sites for the reaction, functionalization, and reduction, which enables further modifications on the absorbent material. In addition, the pristine properties of rGO can be retained in 3D rGO aerogels,^27^ while aerogel structure enables higher specific surface area, lower bulk density, and superior electrical conductivity compared to rGO sheets.^27,28^ The 3D structure of the GO/rGO-based aerogels can be achieved using thermal cross-linking^29^ or chemical cross-linking. Various chemicals have been used to obtain graphene-oxide-based aerogels via chemical cross-linking.^30−33^ The absorption capacities of graphene-oxide-based aerogels have also been examined in the literature. For example, Wu et al.^34^ produced graphene oxide/polyimide aerogels achieving absorption capacities of 14.6–85 g/g using polyimide precursor and GO dispersions. Zhang et al.^35^ prepared graphene aerogels with poly(vinyl alcohol) as the cross-linking agent and ethylene diamine as the reducing agent. Resulting aerogels exhibited absorption capacities within the range of ∼115–285 g/g for various oils. In another study conducted by Che et al.,^36^ methyltriethoxysilane and HI were used along with GO for the synthesis of aerogels. They reached remarkable absorbance capacities of up to 620 mg/mg. In this study, we aimed to prepare graphene oxide and dopamine-based aerogels eliminating the high-temperature requirements or durations.

In the structure of dopamine (DOPA), the catechol structure and amine group, as a good adsorbent,^37^ exist together. Therefore, in the literature, hydrothermal methods have been utilized to obtain ultralight, three-dimensional, and nitrogen-doped graphene aerogels using DOPA.^38−40^ Reaction temperature varied from 85 °C^39^ to 180 °C^31^ for a duration of 12 h. Additional reduction steps in an inert atmosphere were also performed, and both of the aerogels showed efficient space utilization toward oils/organics uptake with maximum absorption capacities of 156 and 282.9 g/g, respectively. Effect of vapor–liquid deposition was also studied after the hydrothermal reaction at 120 °C for 12 h, using 1H,1H,2H,2H-perfluorooctyltriethoxysilane (PFOES).^40^ Absorption capacities for various oils and organics were obtained in the range of 110–230 g/g with superior recyclability.

Herein, we propose a simple, green, efficient, and sustainable production strategy for fabricating recyclable 3D aerogels based on GO and DOPA. Our approach utilizes water as a green solvent, and the production method requires lower energy (lower temperatures and durations) yet yields aerogels with higher absorption capacities compared to earlier studies. The use of DOPA as the cross-linker in obtaining GO-based aerogels enabled not only 3D network formation but also better absorption capacities. The effects of GO and DOPA concentrations on the morphologies and oil and organic absorption properties of aerogels were also evaluated. The composite aerogels exhibited superior absorption capacities toward oils/organics, environmental stability, mechanical durability, and reusability. These aerogels are very promising materials for efficient oil absorption and wastewater remediation.

## 2 Materials and Methods

### 2.1 Materials

Graphite (lateral size ∼300 μm) was kindly supplied by Asbury Carbons. Potassium permanganate (KMnO_4_, Yenilab) was used as an oxidizing agent. Sulfuric acid (H_2_SO_4_, Honeywell), orthophosphoric acid (H_3_PO_4_, VWR Chemicals), acetone (technical grade, VWR Chemicals), hydrochloric acid (HCl, fuming 37%, Merck), and hydrogen peroxide (H_2_O_2_, 30%, Merck) were used in graphene oxide production. Dopamine hydrochloride (DOPA, Alfa Aesar) was used in aerogel production. Dichloromethane, ethanol, toluene, and chloroform were obtained from Sigma-Aldrich and used in the absorption tests. All of the chemicals were used as received.

### 2.2 Synthesis of Graphite Oxide

Graphite oxide was synthesized using the Tour method^41^ with some regulations in the temperatures. Briefly, 1.2 g of graphite flakes (1 wt equivalent) were mixed with 7.2 g of KMnO_4_ (6 wt equivalent), and 160 mL of H_2_SO_4_/H_3_PO_4_ mixture having a volume ratio of 9:1 was slowly added onto the solid mixture under vigorous stirring in an ice–water bath to avoid a sudden increase in the temperature. The reaction was then stirred for 12 h in an oil bath at 50 °C. The reaction mixture was allowed to cool down to room temperature. In an ice bath, 160 mL of an ice–water mixture was added onto the reaction mixture. H_2_O_2_ was added along with ice–water mixture gradually until obtaining bright yellow color, in order to remove the excess KMnO_4_. In the light of studies^41,42^ in the literature, 3.4 wt % HCl (3×) and acetone–ethanol mixtures (4×) were utilized in the purification.

### 2.3 Preparation of GO/D Aerogels

**Figure 1:**
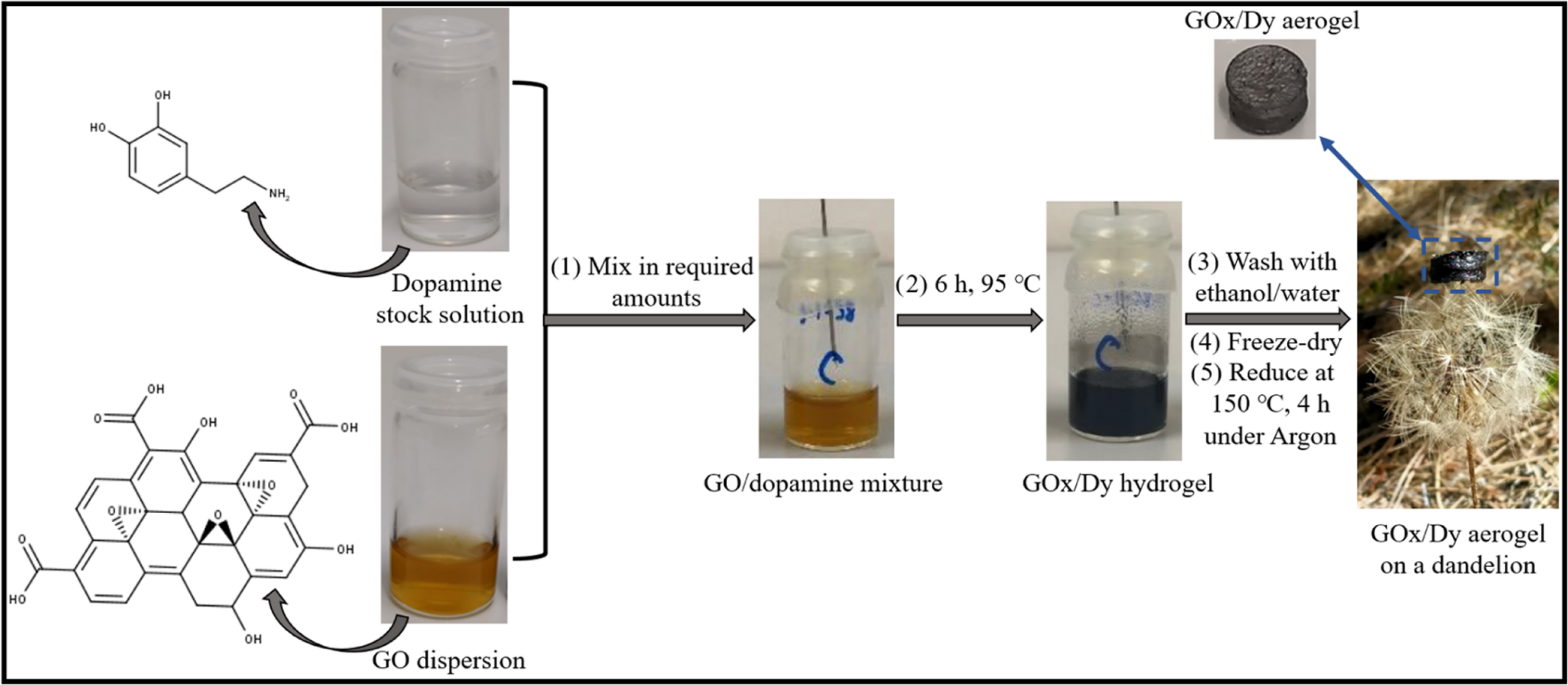
Preparation of the GO*x*/D*y* aerogels.

Stock solutions of DOPA and GO dispersions were prepared in water. Two milliliters of reaction mixtures were prepared by mixing desired amounts of these stock solutions. In order to obtain aerogels with high sorption capacity, precursor solutions were prepared at different DOPA (0.5, 1.0, 1.5 mg/mL) and GO (3, 4, 5 mg/mL) concentrations. The mixtures were then sonicated to eliminate the bubbles, since they disrupt the morphology of the aerogels. They were transferred into an oven at 95 °C for 6 h to obtain precursor hydrogels. Subsequent washing with ethanol/water mixtures and finally with water was performed to ensure the removal of unreacted chemicals (rGO, DOPA, and pDA) from the 3D hydrogel network. UV–vis analyses were used after each wash until there is no trace of unreacted chemicals. After freeze-drying, they were subjected to Ar flow for 15 min and finally reduced at 150 °C for 4 h to obtain polydopamine and reduced GO-based aerogels. Resulting aerogels were named GO*x*/D*y*, *x* and *y* being the GO and DOPA concentrations in the precursor solutions, respectively. The procedure for the aerogel preparation is provided below in Figure 1 along with the chemical structures of the precursors. One of the GO*x*/D*y* aerogels was put on the top of a dandelion to show the lightness of the aerogel. Dopamine solutions were subjected to the same experimental conditions as given in Figure S1a, but gelation could not be observed. GO dispersions were also used as the control group; however, hydrogel formation could not be attained without dopamine addition under the given experimental conditions as provided in the Supporting Information, Figure S1b.

### 2.4 Absorption Experiments

Weighed aerogels were immersed into 15 mL of acetone, chloroform, dichloromethane, ethanol, toluene, diesel oil, water, or sunflower oil for 2 h to reach saturation. Wet gels were weighed, after gentle blotting with a paper towel, and their weight degree of swelling was calculated using the following formula. The graphs representing the weight degree of swelling were constructed with error bars representing the standard deviations of three replicates.
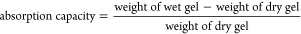


### 2.5 Reusability and Release Studies

Reusability of the samples was examined using chloroform as the absorbate. Loaded chloroform was naturally evaporated at room temperature. Recyclability of the aerogels was assessed by repeating the absorption–evaporation cycles and examining the absorption efficiency. Since sunflower oil is one of the most commonly used oils in domestic use, release studies were conducted using sunflower oil as the absorbate. After 2 h of immersion into sunflower oil, samples were taken out and weighed at different time intervals to assess whether the aerogels had a stable absorption or not.

### 2.6 Characterizations

Attenuated total reflection Fourier transform infrared spectroscopy (ATR-FTIR) in the range 4000–500 cm^–1^ with 64 scans (PerkinElmer Spectrum Two) was used to assess the functional groups on GO and aerogels. Ultraviolet visible spectroscopy (UV–vis) was conducted on GO dispersion in water using Shimadzu UV-2550 in the wavelength range of 200–800 nm. Atomic force microscopy (AFM) analysis was conducted on GO coated onto Si wafer, using Veeco Multimode V AFM on tapping mode. The thermal behavior of the samples was investigated using Shimadzu DTG-60H under a N_2_ atmosphere. Morphological changes in the aerogels were evaluated with scanning electron microscopy (SEM), Tescan Vega3.

## 3 Results and Discussion

### 3.1 Graphite Oxide Synthesis

To develop graphene-oxide-based aerogels for water remediation, graphene oxide was produced from graphite flakes via the Tour Method.^41^ Strong acids provided intercalation of the graphene layers, while KMnO_4_ oxidized the graphene sheets during reaction. The presence of the oxygen-containing functional groups was verified using ATR-FTIR analysis, Figure S2a. In the spectrum of graphite oxide, peaks at the wavenumbers of 1225, 1715, and 3200 cm^–1^ correspond to C–O–C vibrations, C=O stretching vibrations, and O–H stretching vibrations, respectively.^23,41,43,44^ On the other hand, there was no indication of oxygen-containing functional groups in the spectrum of graphite. GO was then further characterized using UV–vis analysis, Figure S2b. Two main characteristics were observed from the spectrum, a peak around 230 nm and a shoulder around 300 nm, which stands for the π → π* transitions of C=C groups and n → π* transitions of carbonyl groups, respectively. These values are in agreement with peak positions reported in the literature.^41,45^ The thermal stability of graphite oxide was also investigated using TGA, as shown in Figure S2c. In TG curves, graphite displayed a minor weight loss (lower than 0.1%) up to 600 °C. The physically absorbed water from GO, which was about 5%, was released until 100 °C.^23^ The major weight loss was seen between 150 and 200 °C likely due to the removal of oxygen-containing functional groups consistent with previous studies.^23,43^ The reason for the weight loss after in between 210 and 600 °C can be due to the desorption of more stable oxygen-containing groups.^24,45^ The dried solid product at the end of synthesis is graphite oxide, formed by the restacked GO sheets. GO as a one atom thick 2D material can be obtained from graphite oxide by exfoliation within solution or cast on a substrate.^1^ AFM results of GO given in Figure S3 showed that the produced material is GO with a thickness around 1 nm.

### 3.2 Preparation and Characterization of GO/D Aerogels

Following the synthesis of GO, aerogels consisting of GO and DOPA were produced. The mixtures were sealed into cylindrical vials, and reaction proceeded in an oven at 95 °C for 6 h. Another set was prepared without dopamine addition; reduction of GO was visible with the color change from yellow to black; however, gelation was not observed regardless of GO concentration as can be seen from Figure S1b. This indicates that DOPA acted as a cross-linker. During the heating process, in addition to the polymerization of dopamine, reduction and gelation occurred in the vials containing both DOPA and GO. The hydrogels took the cylindrical shape of the containing vials. A color change from yellow (reaction mixture) to black (hydrogels) was obtained, as shown in Figure 1. The color change was an “apparent evidence of the reduction^46^” and attributed to the partial restoration of the conjugation network.^47^ The color change was also regarded as a primary indication of deoxygenation or reduction of GO into rGO in many other studies.^46,48−50^

**Figure 2:**
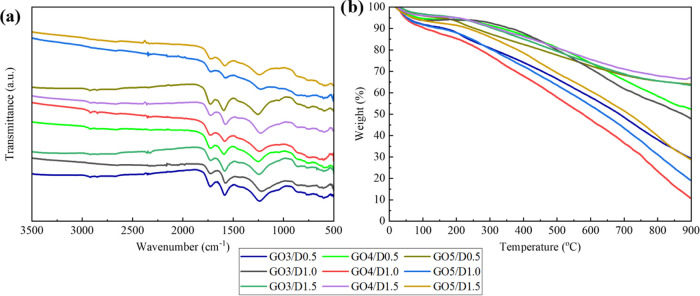
(a) ATR-FTIR analysis and (b) TGA results of GO/D aerogels.

In addition to the color change during incubation at 95 °C for 6 h, deoxygenation of graphene oxide was assessed using ATR-FTIR (Figure 2a). There are several changes for the ATR-FTIR spectrum of the aerogels, which verify the presence of strong interaction between GO and polydopamine.^51^ With the hydrothermal process on the mixture of GO dispersion and DOPA solution, the stretching vibrations of −OH around 3200 cm^–1^ vanished in all of the aerogels.^39^ The disappearance of this band indicates successful reduction according to the literature.^48^ In the ATR-FTIR spectra of the aerogels, the intensity of the peaks denoting oxygen functionalities of GO decreased, supporting the deoxygenation of GO. NH stretching vibrations^39^ were observed at around 1550 cm^–1^, indicating the existence of covalent bonds between polydopamine and GO flakes. To gain more insight into the thermal stability of the aerogels, TGA analysis was also performed. As in GO, the weight loss in Figure 2b up to 100 °C was due to the water absorbed on the surface of the aerogels. The weight loss between 150 and 200 °C was attributed to the removal of oxygen-containing functional groups. When compared with the TGA result of GO provided in Figure S2, it can be seen that the change in the weight percentage within 150–200 °C was more pronounced for GO. More stable functional groups were eliminated with the increase in temperature. The most thermally stable aerogel was observed as GO4/D1.5 among all of the aerogels produced.

**Figure 3:**
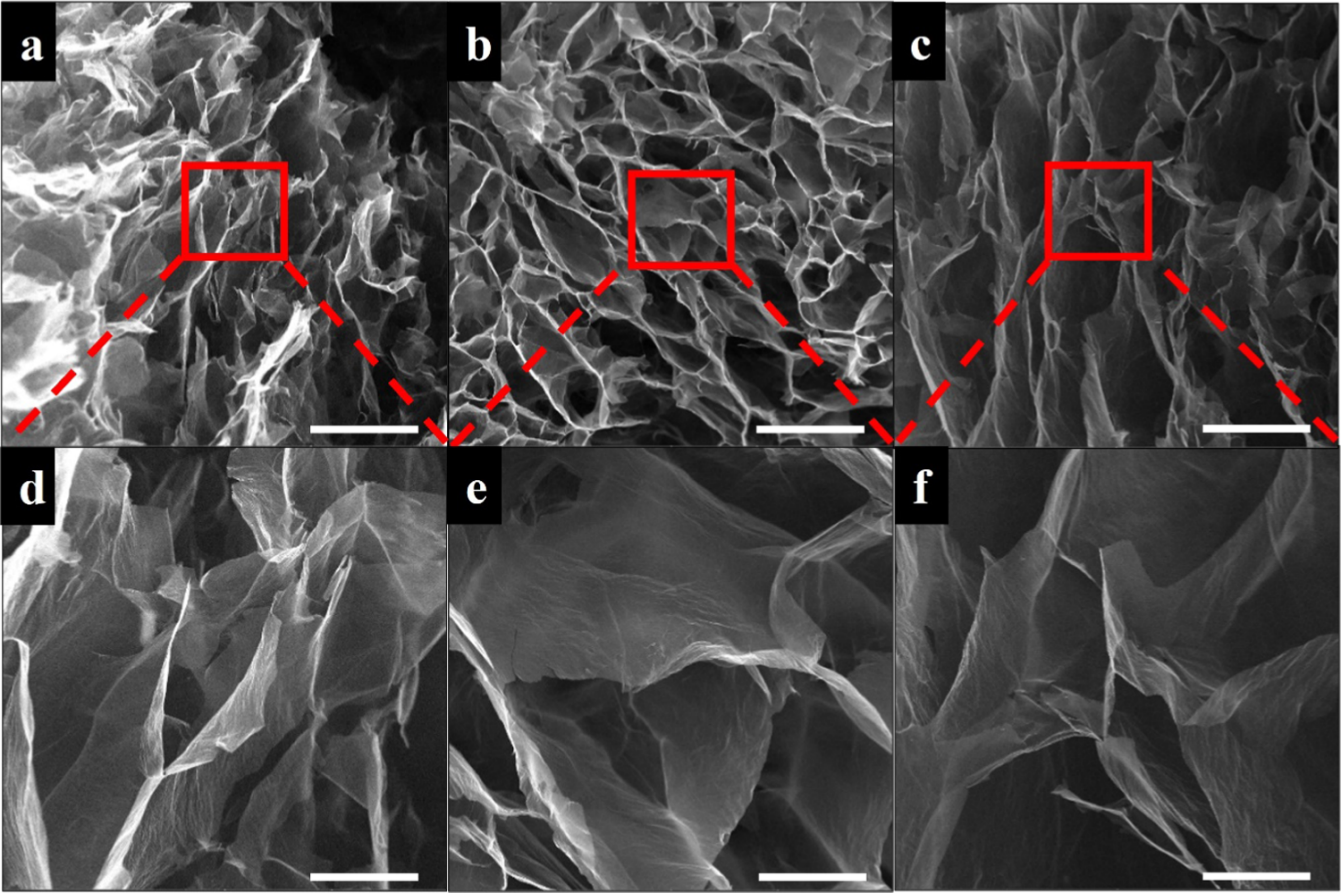
SEM images of GO/D aerogels: (a, d) GO3/D0.5, (b, e) GO3/D1, and (c, f) GO3/D1.5. Scale bar: 200 μm (a–c) and 50 μm (d–f).

**Figure 4:**
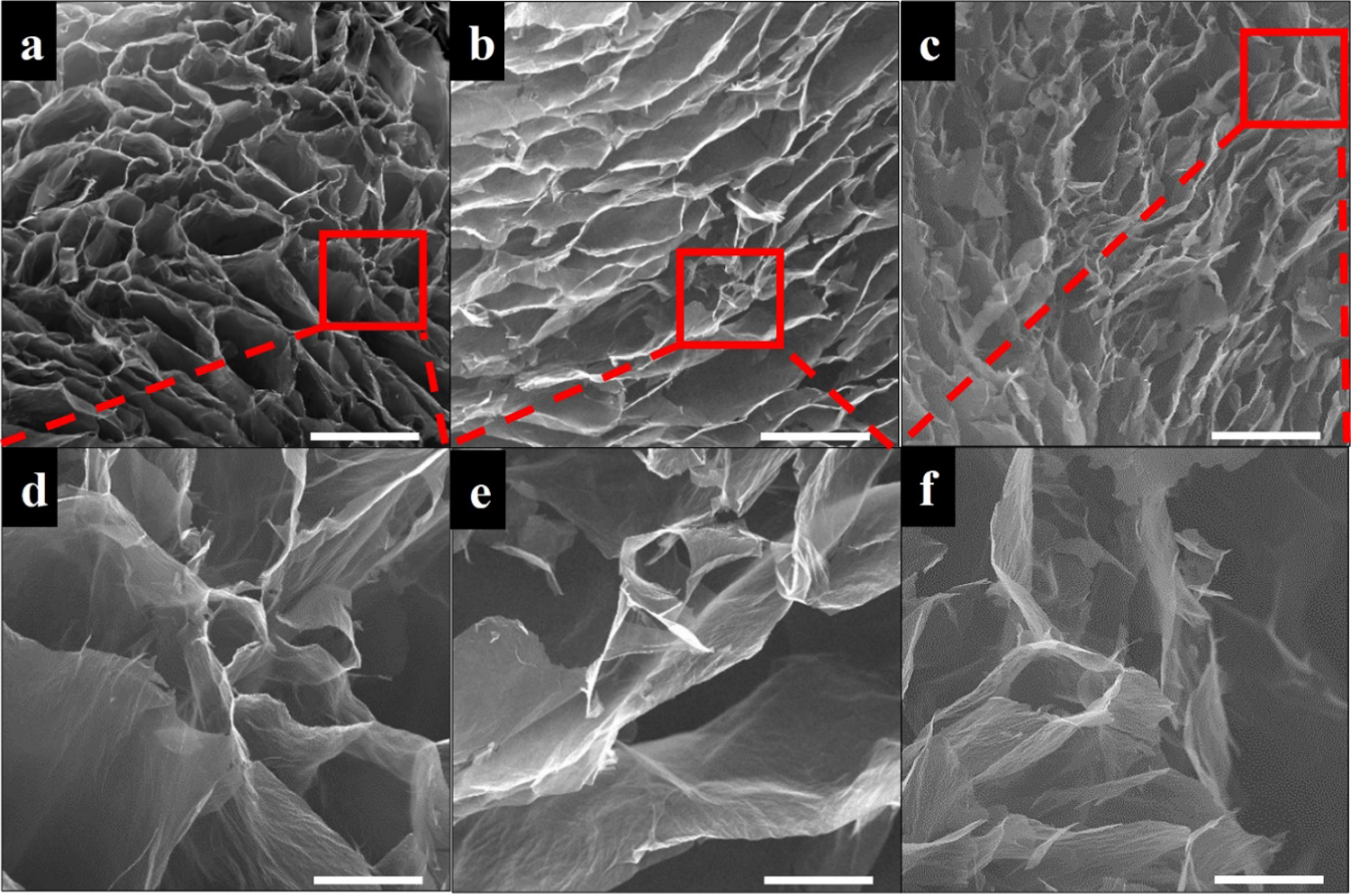
SEM images of GO/D aerogels: (a,d) GO4/D0.5, (b, e) GO4/D1, and (c, f) GO4/D1.5. Scale bar: 200 μm (a–c) and 50 μm (d–f).

**Figure 5:**
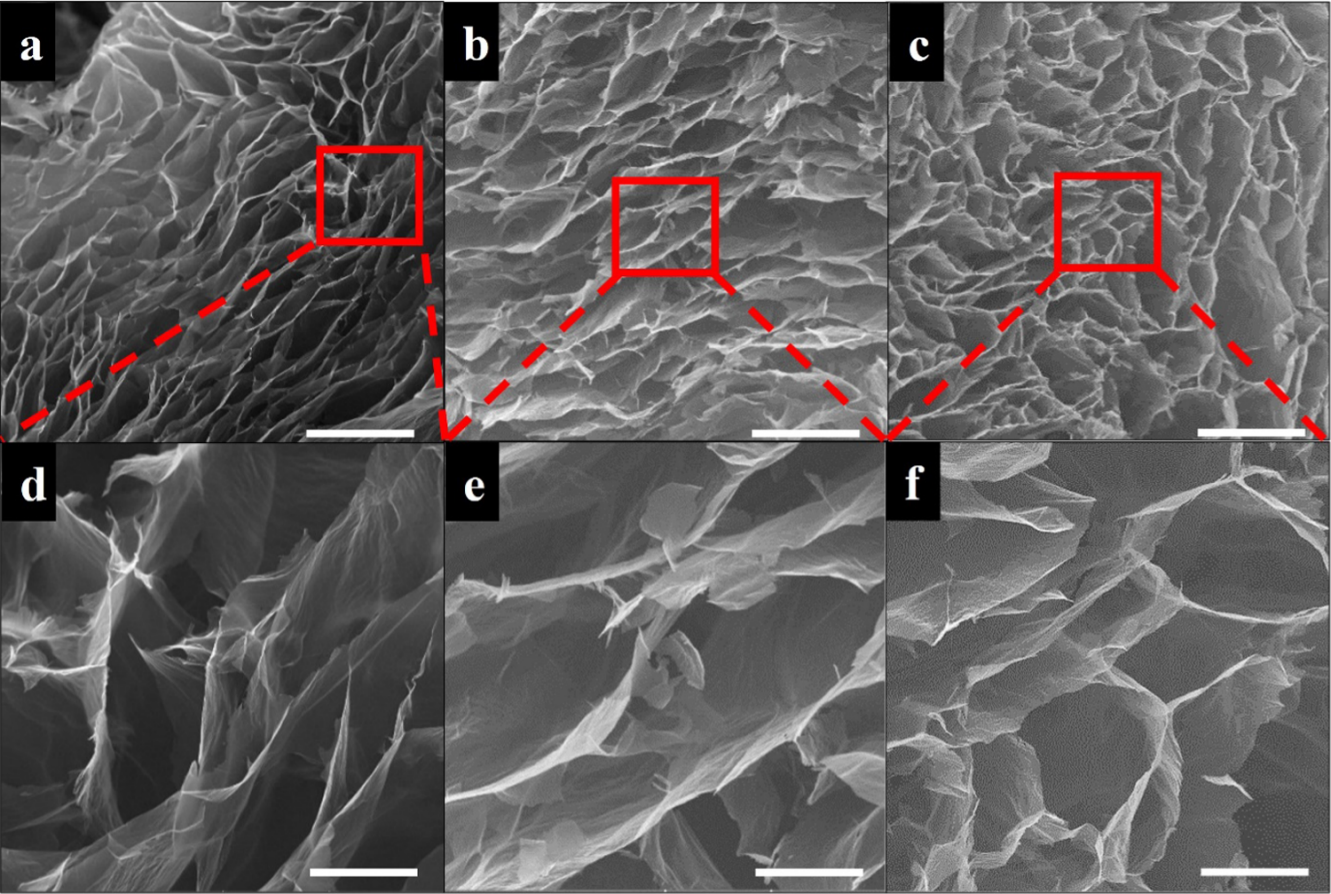
SEM images of GO/D aerogels: (a, d) GO5/D0.5, (b, e) GO5/D1, and (c, f) GO5/D1.5. Scale bar: 200 μm (a–c) and 50 μm (d–f).

In the literature, self-assembly of graphene sheets into 3D structures was attributed to partial overlapping or coalescence of reduced GO nanosheets via noncovalent interactions (hydrogen bonding, π–π interactions, etc.) in the literature.^52^ Although 3D structure could not be obtained when GO sheets were used alone in this study, they were cross-linked with the addition of dopamine to form a 3D and interconnected porous aerogel structure. SEM images of GO/D aerogels given in Figures 3, 4, and 5 clearly show the presence of large and twisted GO sheets having wrinkled surfaces and holes. Dopamine aggregates and oligomers were observed using SEM analysis on the graphene oxide sheets in the literature.^51^ In the SEM images of GO/D aerogels, dopamine aggregates and oligomer formations were not observed, indicating that the washing steps after gelation were successful in removing unreacted precursors. The GO/D aerogels have wide pore size distributions similar to the literature for aerogels containing graphene oxide and dopamine.^39,40,53^ As the concentration of GO increases in the precursor hydrogels, the pore size and pore interconnectivity of the resulting aerogels decrease. Size histograms of SEM images provided in Figure S4 also confirm that pore size decreases with an increase in GO concentration. It was seen that the dopamine concentration increase facilitates the increase in the pore size of the aerogels. However, aerogels having 1.5 mg/mL dopamine concentration lack pore regularity, pores collapsed, and some pore shape distortion occurred.

Before testing the absorption capacities of the aerogels, the porosity of aerogels was determined using the density of graphite in the formula below, ρ_g_, to be 2200 mg/cm^3^. Taking ρ as the density of the aerogel produced (calculated using height, diameter, and weight of the aerogels), the porosities of the aerogels were found to be in the range of 99.80–99.87%, which is in agreement with the literature.^35^
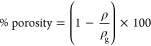


**Figure 6:**
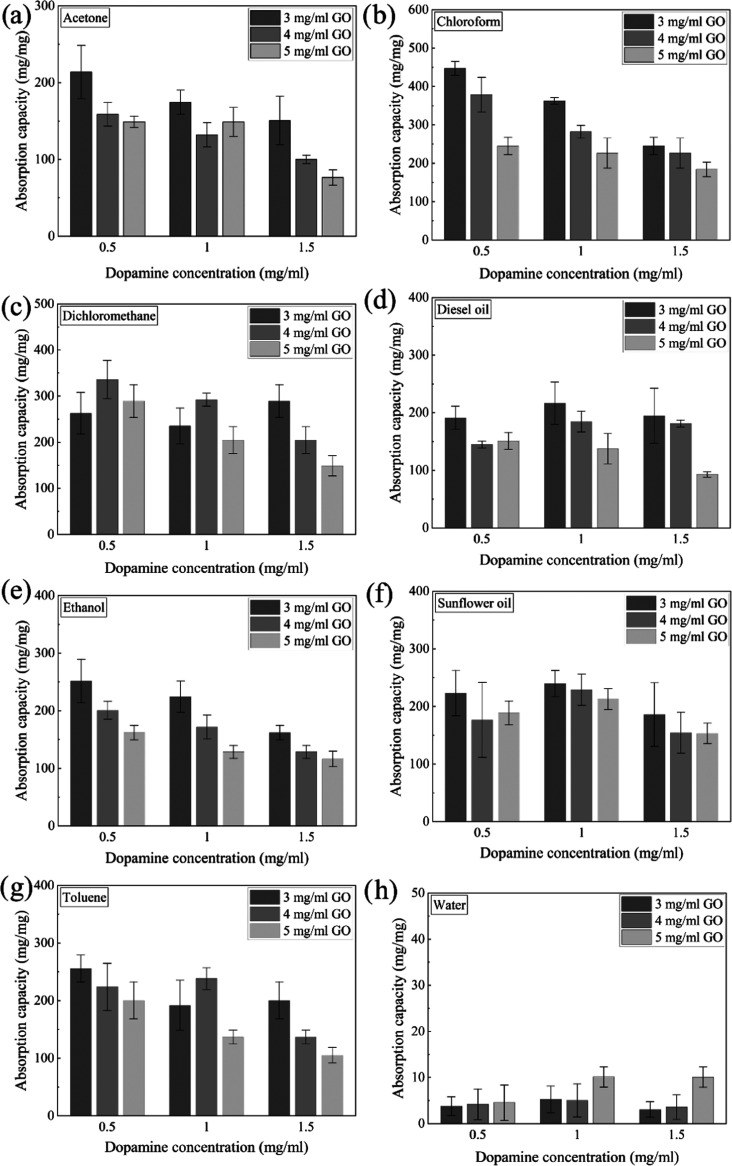
Absorption capacities of aerogels toward (a) acetone, (b) chloroform, (c) dichloromethane, (d) diesel oil, (e) ethanol, (f) sunflower oil, (g) toluene, and (h) water with respect to GO and DOPA concentrations in precursor dispersion.

The absorption capacities of aerogels with varying concentrations of dopamine and GO in the precursor hydrogel are plotted in Figure 6 using the average of three aerogels of the same composition with error bars indicating the corresponding standard deviation. GO/D aerogels exhibited remarkable absorption capacities for several organics (acetone, chloroform, dichloromethane, diesel oil, ethanol, sunflower oil, and toluene) and water. Oxygen-containing functional groups of graphene oxide and amine groups of dopamine along with catechol chemistry could possibly facilitate adsorption of organic liquids onto the surface of aerogels. It can be anticipated that the sorption process starts with the adsorption of organic liquids onto the surface of the aerogel. Then, the liquid diffused into the GO/D aerogel appears to fill out the pores of the 3D structure.

**Figure 7:**
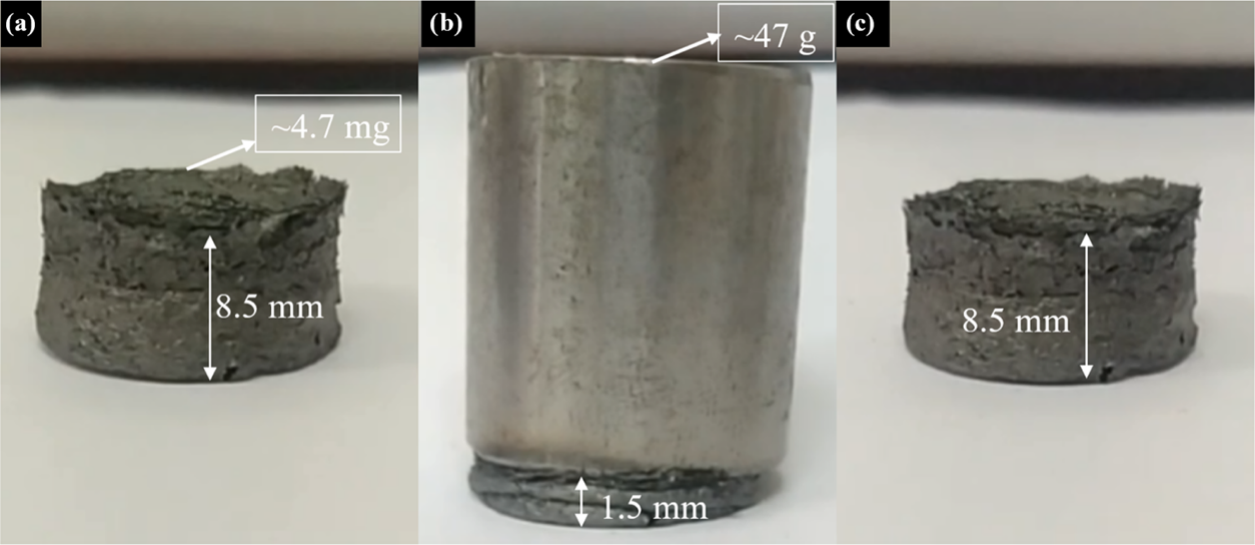
Snapshots of a video showing the GO3/D1 aerogel (a) before, (b) during, and (c) after loading.

It can be seen from Figure 6h that the lowest absorption capacities, about 2–10 mg/mg, were reached using water. According to the figure, an increase in dopamine concentration led to an increase in the absorption of water as well. When the dopamine concentration in the precursor hydrogel was kept at 0.5 mg/mL, the absorption capacities of the aerogels for various organics decreased with increasing GO concentration, which indicates that the higher concentration of GO is less favorable to the exfoliation of GO sheets. Almost all of the organics followed the same trend when the dopamine concentration was increased to 1 or 1.5 mg/mL. According to the SEM images provided, as the concentration of GO was increased, both the pore size and pore connectivity seemed to decrease. Limited diffusion and absorption of the organics due to the disruption of the continuous pore structure may be the cause of such a decrease in the absorption capacities. At the highest dopamine concentration, 1.5 mg/mL, aerogels were brittle regardless of precursor GO concentration. When they were in contact with the absorbate, it became harder to handle them. They were taken out using a spoon and blotted with a paper towel. Therefore, aerogels with the highest dopamine concentration could not be used efficiently and repeatedly for the oil/organic absorption since they lose their mechanical integrity (their surfaces fall off) during/after absorption tests. On the other hand, aerogels with 0.5 and 1 mg/mL DOPA concentrations exhibit elastic behavior under load, preserving their dimensional integrity. As an example, the GO3/D1 aerogel having 4.7 mg weight was subjected to a stainless-steel load having ∼47 g weight, and the aerogel’s behavior was examined in SV1. Snapshots of the video are also presented in Figure 7, and it was seen that the GO3/D1 aerogel can preserve its shape under a load ∼10,000 times of its own weight. For these aerogels (with 0.5 and 1 mg/mL DOPA concentrations), there was no significant weight loss while performing absorption experiments, as well.

In this study, the absorption capacities were in the range of ∼180–445 mg/mg for chloroform, ∼150–335 mg/mg for dichloromethane, ∼93–216 mg/mg for diesel oil, ∼115–250 mg/mg for ethanol, ∼153–230 mg/mg for sunflower oil, and ∼105–255 mg/mg for toluene. As can be seen, the highest absorption capacity of 445 mg/mg was obtained using chloroform and it was higher than that of aforementioned literature studies having absorption capacities ranging from 155^38^ to 280 g/g^39^ for chloroform. Further comparison can be made using Table S1 for the other organics and oils. The highest absorption capacity obtained using GO/D aerogels was listed and compared with the literature studies combining graphene oxide and dopamine to produce aerogels. When the table is examined, it was seen that GO/D aerogels showed higher absorption capacity for various liquids than most of the similar aerogels reported so far.

The oil and organic separation performances of the aerogels from water were also evaluated using a dyed chloroform drop (at the bottom of the beaker) and sunflower oil (on the surface of water) as in SV2. It was shown that all of the chloroform spill can be absorbed from water using a GO*x*/D*y* aerogel less than 3 s after the same aerogel absorbed sunflower oil from the surface of water. There were bubbles coming out, indicating that the air in the aerogel was replaced with chloroform during absorption. Methyl red dyed chloroform and methylene blue dyed water were dropped on the surface of the same type of aerogel to see the surface wettability of the aerogel. It was seen from SV3 that the aerogel can absorb chloroform immediately, whereas water droplets gather on the surface of the aerogel.

**Figure 8:**
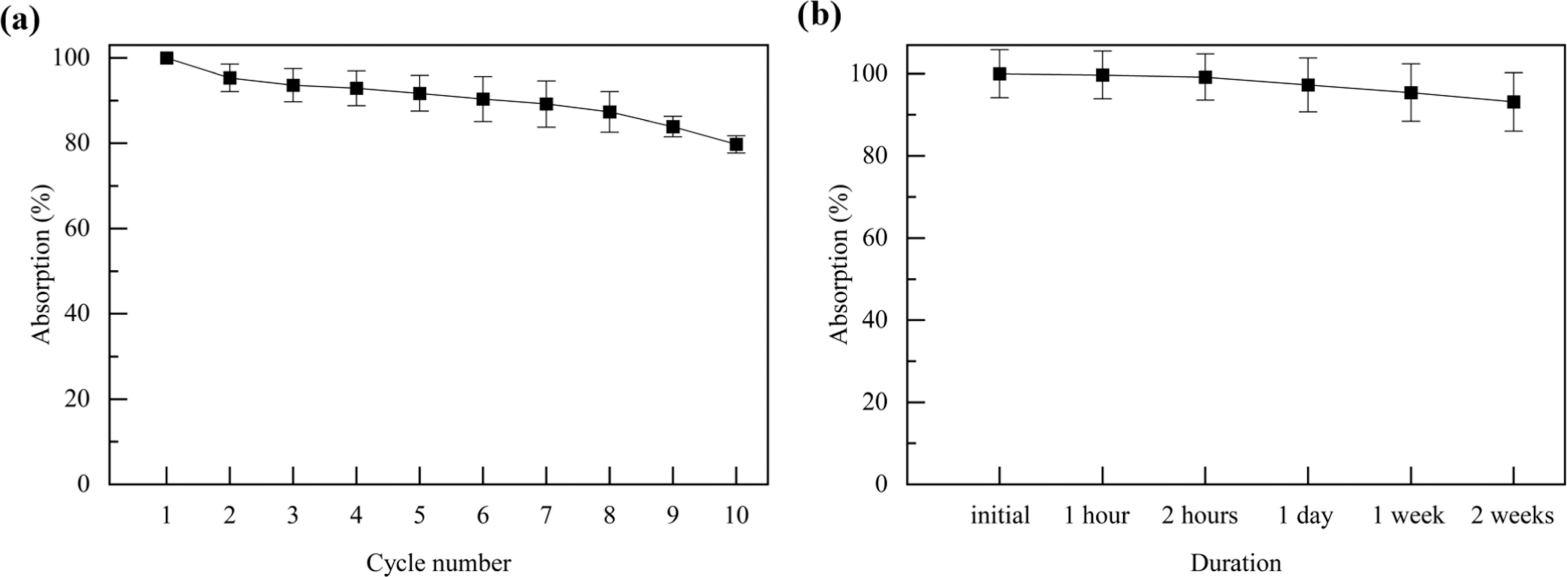
(a) Recyclability and (b) release performance of GO3/D1 aerogels using chloroform and sunflower oil, respectively.

Recyclability/reusability of the aerogels was also examined using three aerogel samples and taking averages to obtain Figure 8a. GO3/D1 aerogels were used in the recyclability tests along with chloroform as the liquid absorbed. Absorbed chloroform was naturally evaporated at room temperature. It was also possible to extract the absorbed chloroform by pressing onto the aerogel, as can be seen from SV4. It was seen that the absorption (%) decreases about 20% after the 10th absorption/desorption cycle, indicating high reusability. Release tests were performed again with three aerogels and taking averages to obtain Figure 8b. Among all of the absorbates used, sunflower oil was the least volatile one. Therefore, it was used in the release tests, and it was seen that the aerogels retained 95% of the absorbate even after 2 weeks.

## 4 Conclusions

Herein, a green, simple, and energy-efficient (less time, lower temperatures) route to produce GO and DOPA-based aerogels was proposed. Use of DOPA as the cross-linker enabled 3D network formation, while nitrogen in DOPA modified aerogels’ surfaces. The effects of the concentrations of DOPA and GO in the precursor solution were examined. The resulting aerogels exhibited low densities between 2.90 and 4.34 mg/cm^3^. Significant improvement was made even with a mild thermal reduction in the absorption capacity of aerogels for oils/organics ranging from 100 mg/mg to 445 mg/mg. The aerogels also exhibited remarkable performances in the reusability tests with 80% efficiency after the 10th cycle with chloroform as the absorbate. In the release experiments, it was found that aerogels can retain 95% of the sunflower oil even after 2 weeks. Based on their separation efficiencies, GO/D aerogels were also found to be promising candidates for water treatment to reduce pollution as a result of organics/oils.

## Supporting Information Available

Photographs of dopamine solutions and GO dispersions having different concentrations before and after thermal treatment, ATR-FTIR, UV–vis, and TGA results of graphite and graphene oxide, AFM results of graphene oxide, comparison of the absorption performance of GO/D aerogels with the literature,comparison of the absorption performance of GO/D aerogels with the literature (PDF)
Behavior of the aerogel under load (SV1) (MP4)
Chloroform and oil absorption from water (SV2) (MP4)
Chloroform and water dropped on the aerogel (SV3) (MP4)
Absorption and release of acetone by pressing onto the aerogel (SV4) (MP4)
