# Supplementary material for: Water-Based Route for Dopamine and Reduced Graphene Oxide Aerogel Production
Source: ACS Omega. 2023 Nov 29;8(49):46728–37. doi: 10.1021/acsomega.3c05955 (PMC10720007; doi:10.1021/acsomega.3c05955)
Supplement: Supplementary file 1 — ao3c05955_si_001.pdf [file ao3c05955_si_001.pdf]

Supporting Information

A Water Based Route for Dopamine and Reduced Graphene Oxide Aerogel Production

Öznur Kavak [a], Barış Can [a,b], Erhan Bat [a]*

[a]Department of Chemical Engineering, Middle East Technical University, Ankara 06800, Turkey

Corresponding Author

*E-mail: bat@metu.edu.tr. Tel.: +90312 210 2634. Fax: +90312 210 2600.

[b] Current address: Norm Coating, İzmir 35620, Turkey

Supporting Information Contents:

Figure S1: Photographs of (a) dopamine solutions and (b) GO dispersions having different concentrations before and after thermal treatment.

Figure S2: Characterization results of graphite and graphene oxide (a) ATR-FTIR spectra, (b) UV-vis spectrum, and (c) TGA thermograms.

Figure S3: AFM results of graphene oxide.

Figure S4: Size histograms of aerogels with DOPA concentration of (a-c) 0.5, (d-f) 1, and (g-i) 1.5 mg/ml.

Table S1: Comparison of absorption performance of GO/D aerogels with literature.

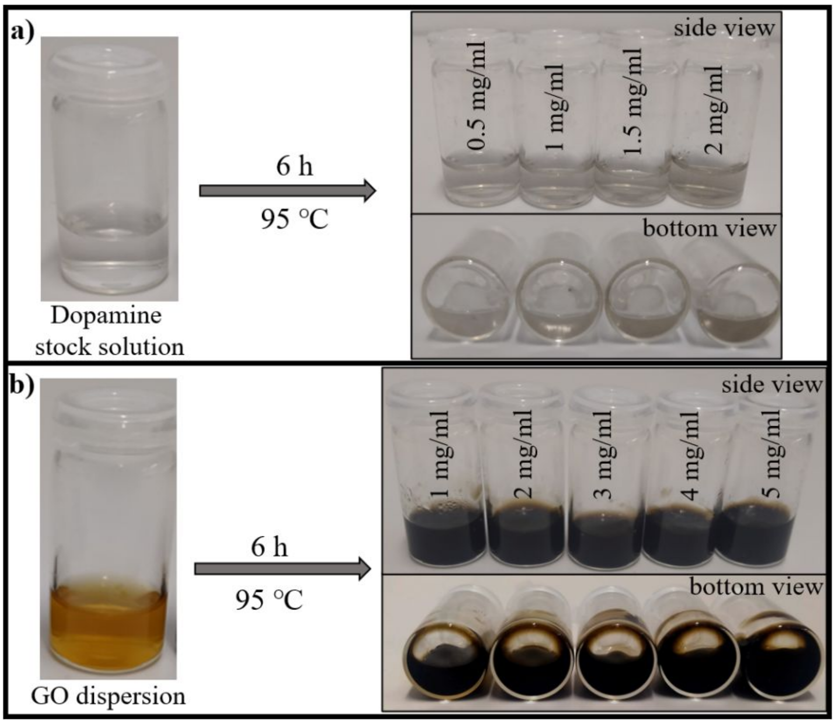

Figure S1: Photographs of (a) dopamine solutions and (b) GO dispersions having different concentrations before and after thermal treatment.

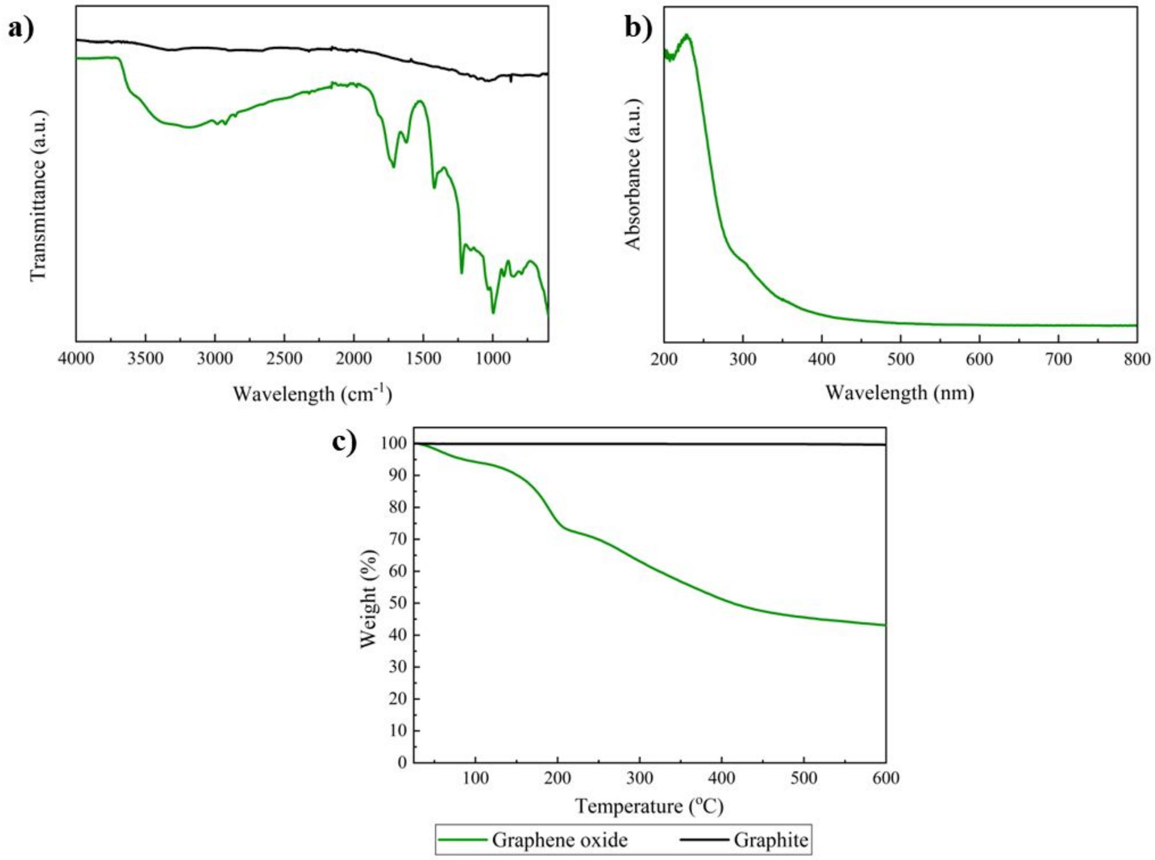

Figure S2: Characterization results of graphite and graphene oxide (a) ATR-FTIR spectra, (b) UV-vis spectrum, and (c) TGA thermograms.

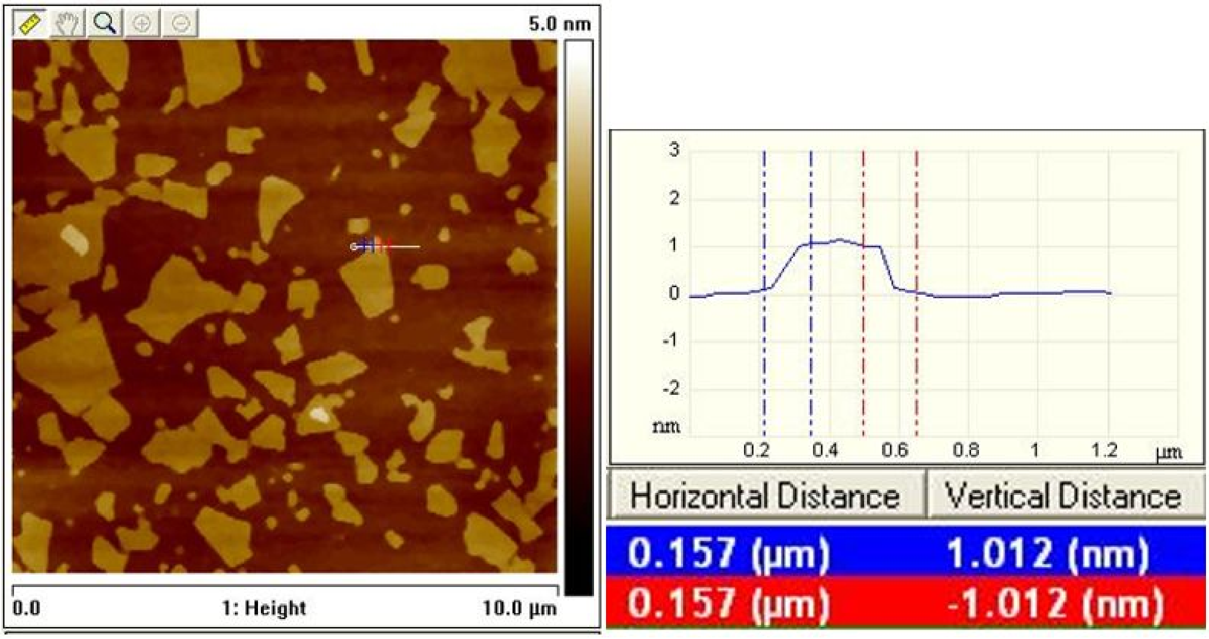

Figure S3: AFM results of graphene oxide.

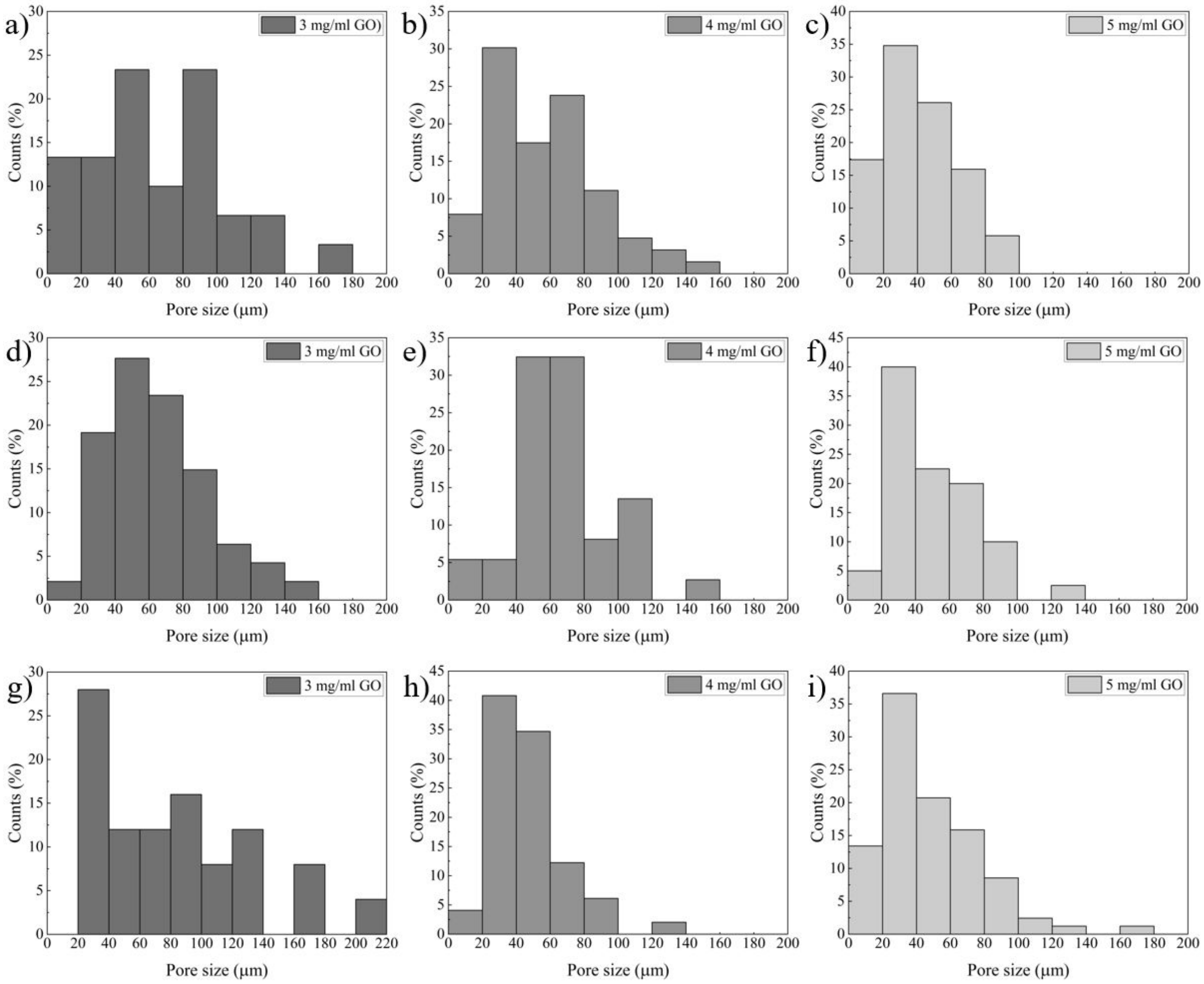

Figure S4: Size histograms of aerogels with DOPA concentration of (a-c) 0.5, (d-f) 1, and (g-i) 1.5 mg/ml.

Table S1: Comparison of absorption performance of GO/D aerogels with literature.

Year	Reference	Results for the absorption/adsorption performance
2013	[1]	Vegetable oil:43 g/g, chlorobenzene:35 g/g, hexane:25 g/g, paraffin oil:37 g/g, toluene:37 g/g, cyclohexane:35 g/g, gasoline:38 g/g
2014	[2]	Gasoline: 120 g/g, soybean oil:105 g/g, pump oil:115 g/g, olive oil:115 g/g, crude oil:100 g/g, diesel oil: 95 g/g, decane: 90 g/g, dodecane:100 g/g, octane: 95 g/g, hexane:65 g/g, chloroform:155 g/g
2016	[3]	Petrol:125 g/g, petroleum ether:140 g/g, n-hexane:160 g/g, ethanol:165 g/g, vacuum pump oil:170 g/g, diesel:180 g/g, dichloromethane:180 g/g, toluene:215 g/g, chloroform:285 g/g
2017	[4]	Ethanol:20 g/g, gasoline:16 g/g, acetone:15 g/g, diesel:13 g/g, hexane:11 g/g, toluene:8 g/g
2018	[5]	n-hexane:110 g/g, octane:120 g/g, hexadecane:145 g/g, toluene:150 g/g, chloroform:230 g/g, gasoline:140 g/g, diesel oil:160 g/g, crude oil:125 g/g, engine oil:200 g/g, peanut oil:215 g/g
2019	[6]	Crude oil:110 g/g, gasoline:140 g/g, diesel oil:130 g/g, engine oil:180 g/g, peanut oil:200 g/g, 1,2-dichlorobenzene:220 g/g, n-hexane:95 g/g, octane:100 g/g, hexadecane:125 g/g, chloroform:205 g/g
2023	This study	Acetone: 214 g/g, chloroform: 447 g/g, dichloromethane: 263 g/g, diesel oil: 216 g/g, ethanol: 252 g/g, sunflower oil: 165 g/g, toluene: 255 g/g, water: 10.1 g/g

REFERENCES

[1] C. Cheng *et al.*, "Biomimetic assembly of polydopamine-layer on graphene: Mechanisms, versatile 2D and 3D architectures and pollutant disposal," *Chem. Eng. J.*, vol. 228, pp. 468–481, 2013, doi: 10.1016/j.cej.2013.05.019.

[2] X. Song, L. Lin, M. Rong, Y. Wang, Z. Xie, and X. Chen, "Mussel-inspired, ultralight, multifunctional 3D nitrogen-doped graphene aerogel," *Carbon N. Y.*, vol. 80, pp. 174–

182, 2014, doi: 10.1016/j.carbon.2014.08.054.

[3] L. Li, B. Li, and J. Zhang, “Dopamine-mediated fabrication of ultralight graphene aerogels with low volume shrinkage,” *J. Mater. Chem. A*, vol. 4, pp. 512–518, 2016, doi: 10.1039/C5TA08829A.

[4] N. Cao *et al.*, “Facile synthesis of fluorinated polydopamine/chitosan/reduced graphene oxide composite aerogel for efficient oil/water separation,” *Chem. Eng. J.*, vol. 326, pp. 17–28, 2017, doi: 10.1016/j.cej.2017.05.117.

[5] S. Yang, C. Shen, L. Chen, C. Wang, M. Rana, and P. Lv, “Vapor–Liquid Deposition Strategy To Prepare Superhydrophobic and Superoleophilic Graphene Aerogel for Oil–Water Separation,” *ACS Appl. Nano Mater.*, vol. 1, pp. 531–540, 2018, doi: 10.1021/acsanm.7b00027.

[6] H. Wang, C. Wang, S. Liu, L. Chen, and S. Yang, “Superhydrophobic and superoleophilic graphene aerogel for adsorption of oil pollutants from water,” *RSC Adv.*, vol. 9, pp. 8569–8574, 2019, doi: 10.1039/c9ra00279k.
